# PDGFR-β^+^ fibroblasts deteriorate survival in human solid tumors: a meta-analysis

**DOI:** 10.18632/aging.202952

**Published:** 2021-05-03

**Authors:** Guoming Hu, Liming Huang, Kefang zhong, Liwei Meng, Feng Xu, Shimin Wang, Tao Zhang

**Affiliations:** 1 Department of General Surgery (Breast and Thyroid Surgery), Shaoxing People’s Hospital, Shaoxing Hospital, Zhejiang University School of Medicine, Zhejiang 312000, China; 2 Department of Nephrology, Shaoxing People’s Hospital; Shaoxing Hospital, Zhejiang University School of Medicine, Zhejiang 312000, China; 3 Department of General Surgery III, Affiliated Hospital of Shaoxing University, Zhejiang 312000, China

**Keywords:** tumor-infiltrating PDGFR-β+ fibroblasts, worse prognosis, solid tumor, meta-analysis

## Abstract

Fibroblasts are a highly heterogeneous population in tumor microenvironment. PDGFR-β^+^ fibroblasts, a subpopulation of activated fibroblasts, have proven to correlate with cancer progression through multiple of mechanisms including inducing angiogenesis and immune evasion. However, the prognostic role of these cells in solid tumors is still not conclusive. Herein, we carried out a meta-analysis including 24 published studies with 6752 patients searched from PubMed, Embase and EBSCO to better comprehend the value of such subpopulation in prognosis prediction for solid tumors. We noted that elevated density of intratumoral PDGFR-β^+^ fibroblasts was remarkably associated with worse overall survival (OS) and disease-free survival (DFS) of patients. In subgroup analyses, the data showed that PDGFR-β^+^ fibroblast infiltration considerably decreased OS in non-small cell lung cancer (NSCLC), breast and pancreatic cancer, and reduced DFS in breast cancer. In addition, increased number of PDGFR-β^+^ fibroblasts appreciably correlated with advanced TNM stage of patients. In conclusion, PDGFR-β^+^ fibroblast infiltration deteriorates survival in human solid tumors especially in NSCLC, breast and pancreatic cancer. Hence, they may offer a practicable prognostic biomarker and a potential therapeutic strategy for these patients.

## INTRODUCTION

Tumor microenvironment (TME) is linked closely with the initiation, promotion and progression of cancer. Fibroblasts, the major composition of cancer stroma, are often activated by various stimuli such as some cytokines secreted by tumor cells in the TME [1]. Increasing research has documented that tumor-infiltrating fibroblasts could facilitate cancer progression through a multitude of mechanisms including inducing angiogenesis and immune suppression [2].

PDGFR-β, a major regulatory protein for mesenchymal cells such as fibroblasts and mesangial cells, is becoming a pivotal controller of these cells in TME of numerous malignancies including breast and prostate cancer [3]. Recently, many studies have demonstrated that PDGFR-β was frequently upregulated in fibroblasts in tumor stroma [4], and could well represent the activation status of fibroblasts [5]. In the last decades, although a great number of researchers have investigated the association between intratumoral PDGFR-β^+^ fibroblasts and prognosis in human solid tumors, their results were controversial [6]. It needs further investigation, in addition, the potential of intratumoral PDGFR-β^+^ fibroblasts as a practicable prognostic biomarker and targeted strategy is required to be explored.

In this study, we carried out a meta-analysis to quantitatively assess the correlation between tumor-infiltrating PDGFR-β^+^ fibroblasts and clinical outcomes in solid tumors, and found that high density of intratumoral PDGFR-β^+^ fibroblasts was remarkably associated with worse overall survival (OS) and disease-free survival (DFS) of patients. In subgroup analyses, PDGFR-β^+^ fibroblast infiltration considerably decreased OS in non-small cell lung cancer (NSCLC), breast and pancreatic cancer, and reduced DFS in breast cancer of patients. Moreover, increased number of PDGFR-β^+^ fibroblasts appreciably correlated with advanced TNM stage of patients. Hence, we may offer a practicable prognostic biomarker and a potential therapeutic strategy for these patients.

## MATERIALS AND METHODS

### Literature search

PubMed, Embase and EBSCO were retrieved to evaluate the PDGFR-β^+^ fibroblast infiltration and clinical outcomes in solid tumors from January 1980 to November 2020. The keywords for searching strategy were: (fibroblasts [Title/Abstract] OR PDGFR-β [Title/Abstract]) AND (tumor [Title/Abstract] OR cancer [Title/Abstract] OR carcinoma [Title/Abstract] OR neoplasms [Title/Abstract]) AND (survival [Title/Abstract] OR prognosis [Title/Abstract]).

### Inclusion and exclusion criteria

Studies included in this meta-analysis should meet the following inclusion criteria: (1) been published as original articles in English; (2) investigated human subjects; (3) tested PDGFR-β^+^ fibroblasts in primary tumor lesions; (4) supplied hazard ratios (HRs), or Kaplan – Meier curves exhibiting the association between PDGFR-β^+^ fibroblasts and OS, and/or DFS.

The exclusion criteria were that studies haven’t been published as research article or full text such as case report, commentary, letter and conference abstract. Studies without sufficient data for hazard ratios (HRs) calculation or detecting fibroblasts in metastatic tissues, or not with marker ‘PDGFR-β’ were also excluded.

### Endpoints

OS and DFS were considered as the primary and second endpoint respectively in this meta-analysis.

### Data extraction

Two authors (GM.H. and KF.Z.) independently extracted data such as number of patients, follow-up time, method applied for quantifying PDGFR-β^+^ fibroblasts as well as the cut-off value for identifying increased density of such subpopulation. OS, DFS and clinicopathological features including primary tumor, lymph node, distant metastasis (TNM) stage as well as tumor differentiation were obtained from the text, tables and Kaplan – Meier curves.

### Quality evaluation

Two authors adopted Newcastle–Ottawa Scale (NOS) [7] to assess the quality of individual research independently, and achieved consensus with the assistant of the third or more authors. Six or above that the study scored was regarded as high quality.

### Subgroup analyses

In this study, the subgroup analyses between PDGFR-β^+^ fibroblasts infiltration and OS or DFS were conducted according to tumor types.

### Statistical analysis

Relevant data were combined into hazard ratios (HRs) for OS, DFS, and odds ratios (ORs) for clinicopathological features such as TNM stage, lymph node metastasis, tumor differentiation with STATA 12.0 respectively based on the random-effect model if statistical heterogeneity was considerable [8], otherwise, the fixed-effect model was adopted [9]. We also used sensitivity test, Begg’s funnel plot and Egger’s analysis [10] to investigate the impact of each research on overall result and publication bias respectively. We considered that there was statistical significance when *P* value was less than 0.05.

## RESULTS

### Search results and characteristics of studies

Flow chart diagram of study selection was stated in Supplementary Figure 1. We finally included 24 researches with 6752 patients in this meta-analysis [11–34], and then assessed these included cohort researches with Newcastle–Ottawa Scale (NOS). Characteristics of researches being appropriate for data integration were exhibited in Table 1 and Supplementary Table 1.

**Table 1:** Features of individual included research.

**Research**	**Year**	**Type of tumor**	**Patients’ No.**	**M / F**	**Median age (range) (year)**	**Cut-off value**	**PDGFR-β^+^ fibroblast: (H/L)**	**TNM stage**	**Median follow-up (months)**	**Clinical outcome**	**Quality score (NOS)**
Park, C.K. et al [11]	2016	Breast cancer	524	0/524	<50: 55.8%; ≥50: 44.2%	≥ 10% of the stroma /HPF	153/489	I - III	NR	OS, DFS	8
Park, S.Y. et al [12]	2015	Breast cancer	642	0/642	≤50: 60.3%; >50: 39.7%	≥ 10% of the stroma /HPF	153/489	I - III	68.3 ± 30.1	OS, DFS	8
Kim, H.M. et al [13]	2016	Malignant phyllodes tumor of breast	16	0/16	47.6 ± 12.9	≥ 30% of the stroma /HPF	5/11	NR	NR	OS, DFS	8
Jung, Y.Y. et al [14]	2015	Breast cancer	642	0/642	≤50: 60.3%; >50: 39.7%	≥ 10% of the stroma /HPF	23/619	I - III	68.3 ± 30.1	OS, DFS	7
Paulsson, J. et al [15]	2009	Breast cancer	289	0/289	64.2 (27, 96)	≥ 10% of stromal fibroblasts /HPF	100/189	I - III	106 (0, 207)	OS, DFS	8
Kilvaer, T.K. et al [16]	2019	NSCLC	513	343/170	<65: 42.5%; ≥65: 57.5%	Score ≥2	202/311	IA - IIIB	NR	OS	7
Kanzaki, R. et al [17]	2018	NSCLC	92	78/14	60.2	≥ 5% of the stroma /HPF	65/27	IA - IV	187 (48, 260)	OS, DFS	8
Donnem, T. et al [18]	2008	NSCLC	335	255/80	67 (28, 85)	Score ≥ 2.5	69/262	I - IIIA	96 (10, 179)	OS	7
Kilvaer, T.K. et al [19]	2018	NSCLC	499	161/338	<65: 42.9%; ≥65: 57.1%	≥ 10% of the stroma /HPF	199/300	IA - IIIA	48.0 (1, 137)	OS	7
Chu, J.S. et al [20]	2013	Hepatic carcinoma	93	77/16	≤50: 33.3%; >50: 66.7%	≥ 50% of stroma /10HPF	18/75	III	(1, 58)	OS	6
Zhang, X.F. et al [21]	2017	Intrahepatic cholangiocarcinoma	41	NR	NR	≥ 20% of the stroma /HPF	33/8	I - IV	15.7 (1.3, 63.2)	OS	6
Chen, L. et al [22]	2011	Hepatic carcinoma	63	59/4	48.9 (30, 73)	≥ 26% of the stroma /HPF	43/20	I - IV	46.7 (40.3, 62.1)	OS, DFS	7
Sayaka, Y. et al [23]	2012	Pancreatic adenocarcinoma	26	18/8	61.5 (45, 81)	NR	13/13	I - IVB	NR	OS	6
Kurahara, H. et al [24]	2016	Pancreatic cancer	120	71/49	≤70: 60.8%; >70: 39.2%	score > 2	59/61	NR	29.2	OS	6
Hagglof, C. et al [25]	2010	Prostate cancer	244	244/0	74 (51, 95)	mean density ≥1.0	66/178	NR	(1, 300)	OS	7
Nordby, Y. et al [26]	2017	Prostate cancer	529	529/0	62 (47, 75)	mean density ≥1.50	262/267	I - IV	148.8 (18, 240)	DFS	6
Frodin, M. et al [27]	2017	Renal cell carcinoma	287	162/125	(37, 89)	NR	144/143	I - IV	NR	OS	8
Shim, M. et al [28]	2015	Renal cell carcinoma	758	536/222	55 (47, 64)	≥ 33% of the stroma /HPF	302/456	I - II	29.5 (21.5, 39.6)	DFS	7
Corvigno, S. et al [29]	2016	Ovarian cancer	154	0/154	60 (22, 84)	≥ 10% of the stroma /HPF	79/75	I - IV	28 (0.03, 162.5)	OS	7
Mezheyeuski, A. et al [30]	2016	Colorectal cancer	372	182/190	(18, 75)	≥ 50% of the stroma /HPF	NR	IV	9 (7.8, 10.2)	OS	7
Yonemori, K. et al [31]	2011	Angiosarcoma	34	9/25	68 (16, 96)	Score ≥1	30/4	I - III	26.7 (0.3, 152.6)	OS	7
Ha, S.Y. et al [32]	2014	Esophageal squamous cell carcinoma	116	112/4	<65: 26.7%; ≥65: 73.3%	≥ 50% of the stroma /HPF	63/53	I - IV	30 (0, 108)	OS, DFS	6
Moreno, L. et al [33]	2013	Ependymoma	24	15/9	(1.5, 64.9)	≥ 50% of the stroma /HPF	7/17	IV	32.3 (2.1, 59.1)	OS	6
Sun, W.Y. et al [34]	2015	Thyroid papillary carcinoma	339	NR	NR	≥ 50% of the stromal cells /HPF	72/267	NR	NR	OS, DFS	6

OS: overall survival; DFS: disease-free survival; TNM, Tumor, Lymph Node, Metastasis; NR: not reported; HPF: high power field. M: male; F: female.

### Meta-analyses

### OS

**Figure 1:**
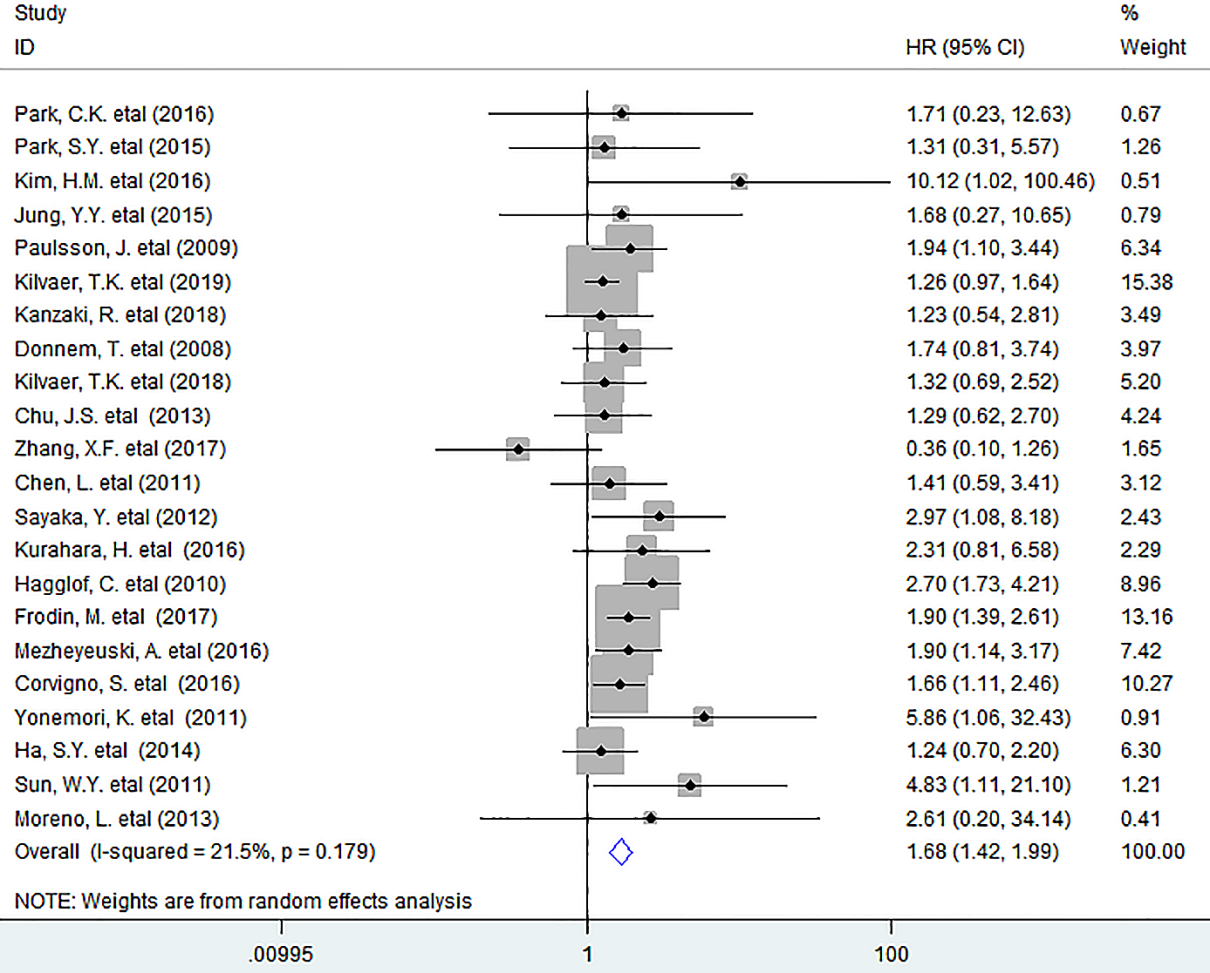
**Forest plots describing HR of the association between PDGFR-β^+^ fibroblast infiltration and OS in solid tumors.** HRs: hazard ratios; OS: overall survival.

In this study, we noted that increased number of tumor-infiltrating PDGFR-β^+^ fibroblasts remarkably reduced OS (HR = 1.68, 95% CI 1.42 to 1.99, *P < 0.001*) in human solid tumors, with little heterogeneity being detected among included researches (*I^2^ = 21.5%, P = 0.179*) (Figure 1).

**Figure 2:**
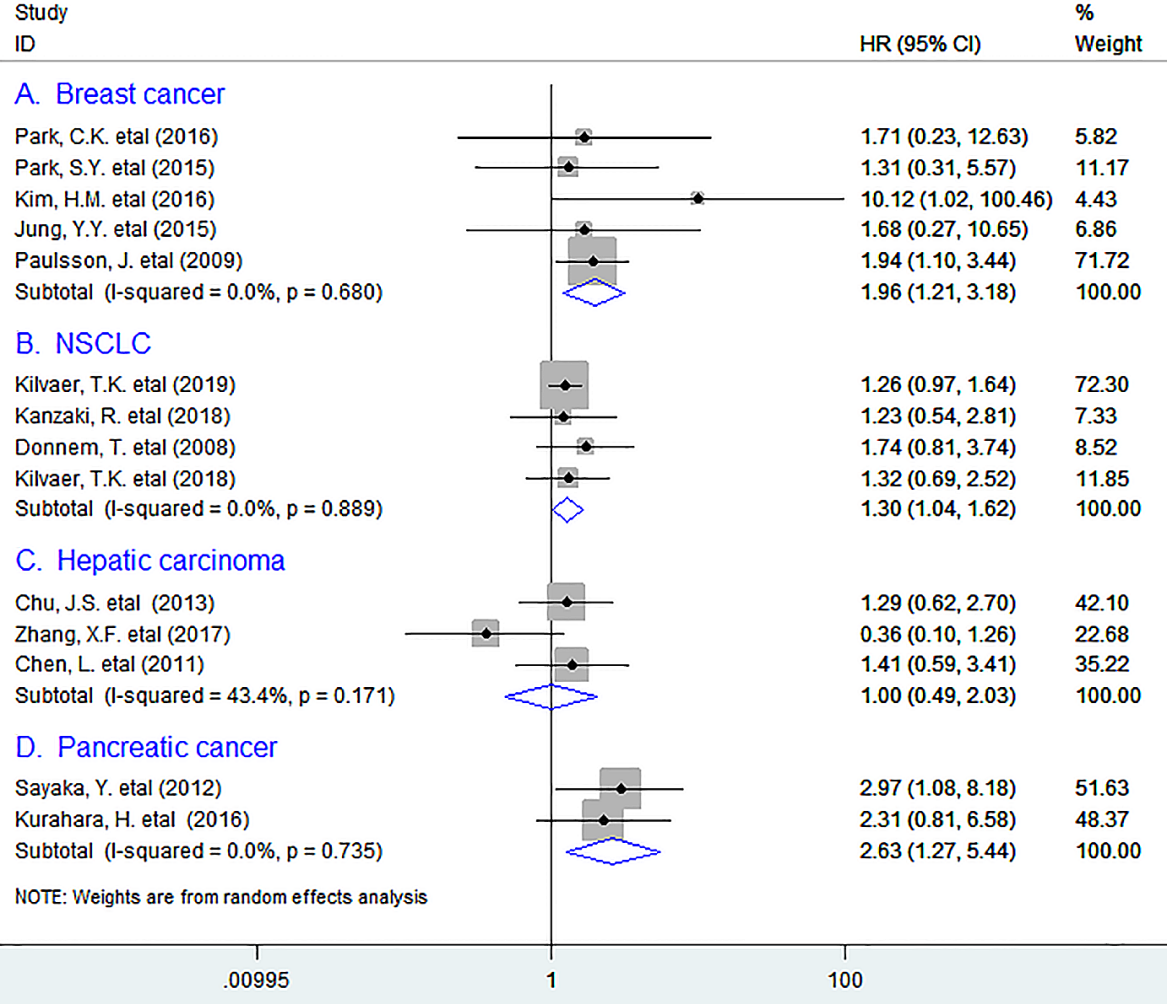
Subgroup analyses describing HRs of the association between PDGFR-β+ fibroblast infiltration and OS in breast cancer (**A**), NSCLC (**B**), Hepatic carcinoma (**C**), and pancreatic cancer (**D**). HRs: hazard ratios; OS: overall survival.

In subgroup analyses based on tumor types, the pooled data indicated that high density of PDGFR-β^+^ fibroblasts within tumor was markedly associated with reduced OS in breast cancer (BC) (HR = 1.96, 95% CI 1.21 to 3.18, *P* = 0.006), with no heterogeneity being observed; Similar results were observed between PDGFR-β^+^ fibroblasts and OS in non-small cell lung cancer (NSCLC) (HR = 1.30, 95% CI 1.04 to 1.62, *P* = 0.021), and pancreatic cancer (PC) (HR = 2.63, 95% CI 1.27 to 5.44, *P* = 0.009).(Figure 2) However, we were unable to obtain a combined result for several types of tumor including ovarian cancer, renal cell carcinoma, colorectal cancer (CRC), esophageal squamous cell carcinoma, angiosarcoma and thyroid papillary carcinoma as there was only one study that supplied sufficient data for such type of tumor.

### DFS

**Figure 3:**
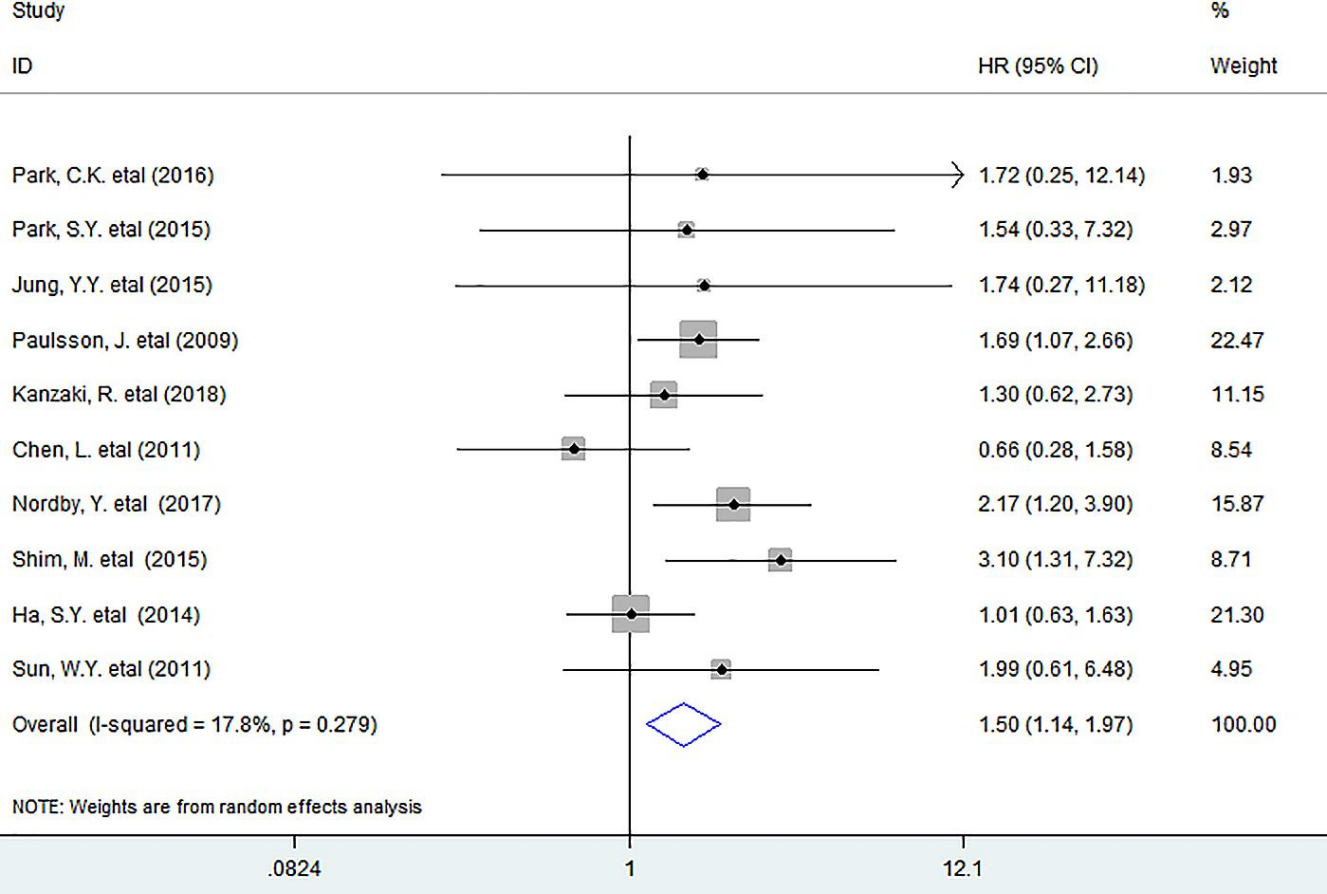
**Forest plots describing HR of the association between PDGFR-β^+^ fibroblast infiltration and DFS in solid tumors.** HRs: hazard ratios; DFS: disease-free survival.

Pooled data showed that the infiltration of PDGFR-β^+^ fibroblasts appreciably decreased DFS (HR = 1.50, 95% CI 1.14 to 1.97, *P* = 0.004) of patients (Figure 3).

**Figure 4:**
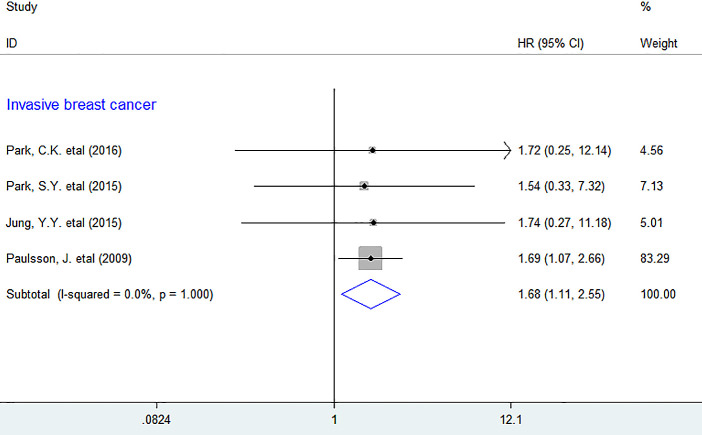
**Subgroup analyses describing HRs of the association between PDGFR-β^+^ fibroblast infiltration and DFS.** HRs: hazard ratios; DFS: disease-free survival.

In subgroup analyses, we discovered that increased number of intratumoral PDGFR-β^+^ fibroblasts was considerably associated with lower DFS in BC (HR = 1.68, 95% CI 1.11 to 2.55, *P* = 0.014), with little heterogeneity being detected (*I^2^ = 0%, P = 1.000*). (Figure 4) However, there was no sufficient data for other types of tumor, so we were unable to obtain the combined result.

In addition, we found that elevated density of those cells was remarkably associated with advanced TNM stage (OR = 0.47, 95% CI 0.25 to 0.86, *P* = 0.015), but not with lymph node metastasis or tumor differentiation of patients (Supplementary Figure 2).

### Sensitivity analyses

Sensitivity analyses revealed that each individual study didn’t have impact on overall result for OS or DFS (Supplementary Figure 3).

### Publication bias

Funnel plot and Egger’s tests indicated that no potential publication bias existed between tumor-infiltrating PDGFR-β^+^ fibroblasts and OS (*P* = 0.305) or DFS (*P* = 0.727) (Supplementary Figure 4).

## DISCUSSION

Although multitudinous researchers have correlated tumor-infiltrating PDGFR-β^+^ fibroblasts and survival in human solid tumors for the past decades, the results were inconsistent even controversial. In this study, we noted that PDGFR-β^+^ fibroblast infiltration significantly decreased survival in solid tumors especially in BC, NSCLC and PC. In addition, increased number of PDGFR-β^+^ fibroblasts remarkably correlated with advanced TNM stage. Hence, we harbor the idea that this is the first to exhibit the important prognostic value of tumor-infiltrating PDGFR-β^+^ fibroblasts in human solid tumors.

We considered that the following evidence can probably explain the negative correlation between intratumoral PDGFR-β^+^ fibroblasts and prognosis of patients. First, tumor-infiltrating fibroblasts can trigger proliferation, survival and invasion of tumor cells by releasing a variety of growth factors, cytokines, chemokines and degradation of extracellular matrix proteins including matrix metalloproteinases (MMPs) (e.g. MMP9) [35, 36]; Second, they can also produce hydrogen peroxide to induce carcinogenesis, promote epithelial-mesenchymal transition of tumor cells, [37] and induce CD73^+^γδTreg cell differentiation via IL-6 secretion thereby facilitating immune suppression [38]. More importantly, *in vivo* experiments have indicated that the activation of PDGFR-β in fibroblasts mediated by its ligand (PDGF-β) can promote the accumulation and expansion of these cells in primary tumor thereby prompting cancer progression [39, 40]. In addition, PDGFR-β^+^ fibroblasts can stimulate tumor growth through inducing angiogenesis by generating proangiogenic factors such as VEGF [41]. Furthermore, they can dampen antitumor immunity and promote cancer immune evasion via secreting immunosuppressive cytokines including TGF-β1 [41], and recruiting MDSCs through CCL2 released in the TME [42]. Hence, it is rational to conclude that the PDGFR-β^+^ fibroblasts are prone to foster tumor progression and decrease survival.

Several limitations existed in the meta-analysis. For example, morphometric analyses applied for assessment of PDGFR-β^+^ fibroblasts in individual included studies were inconsistent. In addition, there was no sufficient data for OS in certain types of tumor, we were therefore unable to obtain pooled results for them.

In conclusion, PDGFR-β^+^ fibroblast infiltration deteriorates survival in human solid tumors especially in NSCLC, breast and pancreatic cancer. They may therefore provide a practicable prognostic biomarker and a potential therapeutic strategy.

### Ethics approval and consent to participate

Ethical approval is not required for this article.

### Availability of data and materials

The datasets supporting the conclusions of this article are included within the article.

## Supplementary Material

Supplementary Figures

Supplementary Table 1
