# Supplementary material for: PDGFR-β+ fibroblasts deteriorate survival in human solid tumors: a meta-analysis
Source: Aging (Albany NY). 2021 May 3;13(10):13693–707. doi: 10.18632/aging.202952 (PMC8202854; doi:10.18632/aging.202952)
Supplement: Supplementary Figures [file aging-13-202952-s001.pdf]

SUPPLEMENTARY FIGURES

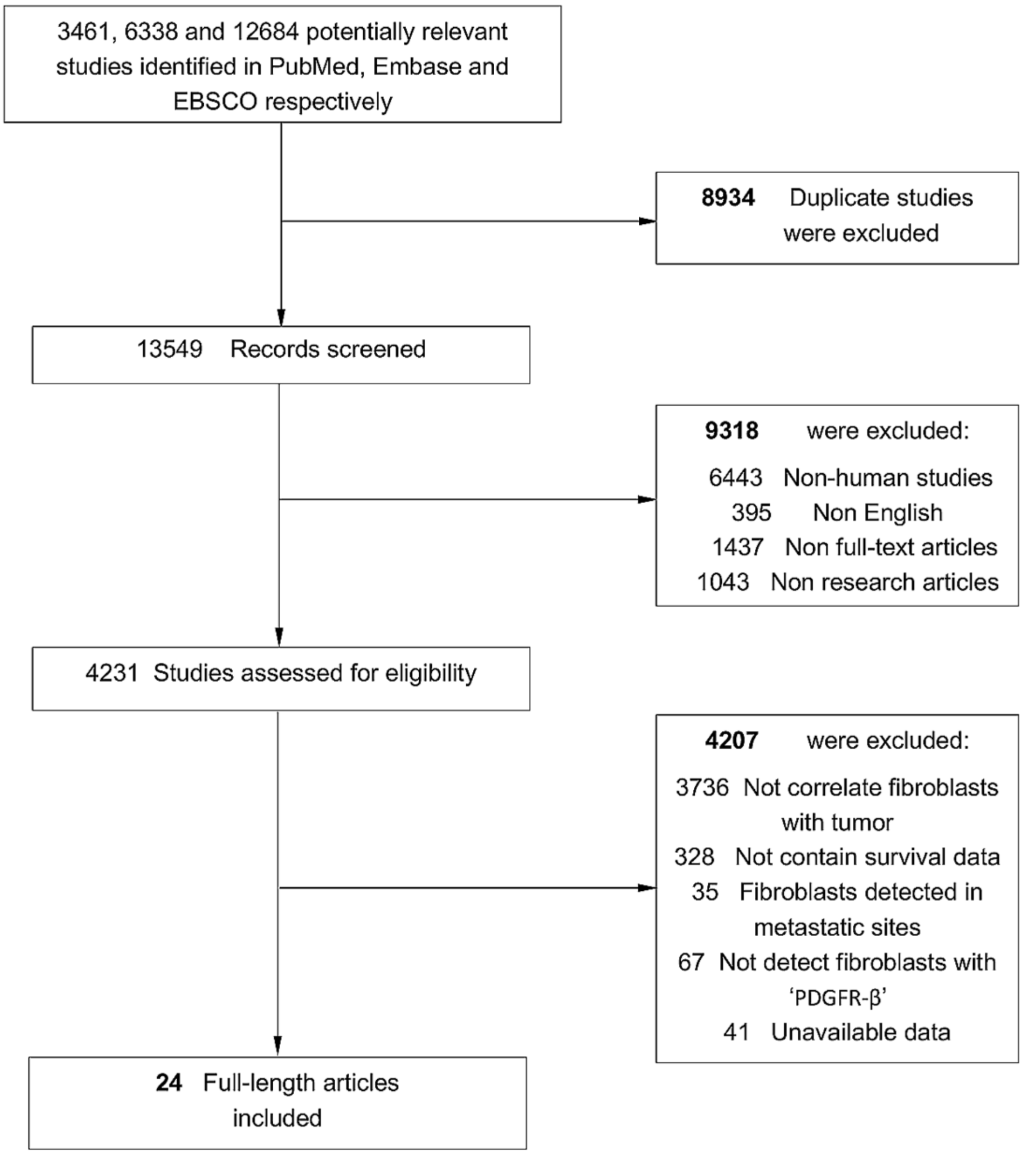

Supplementary Figure 1. Flow chart diagram of study selection.

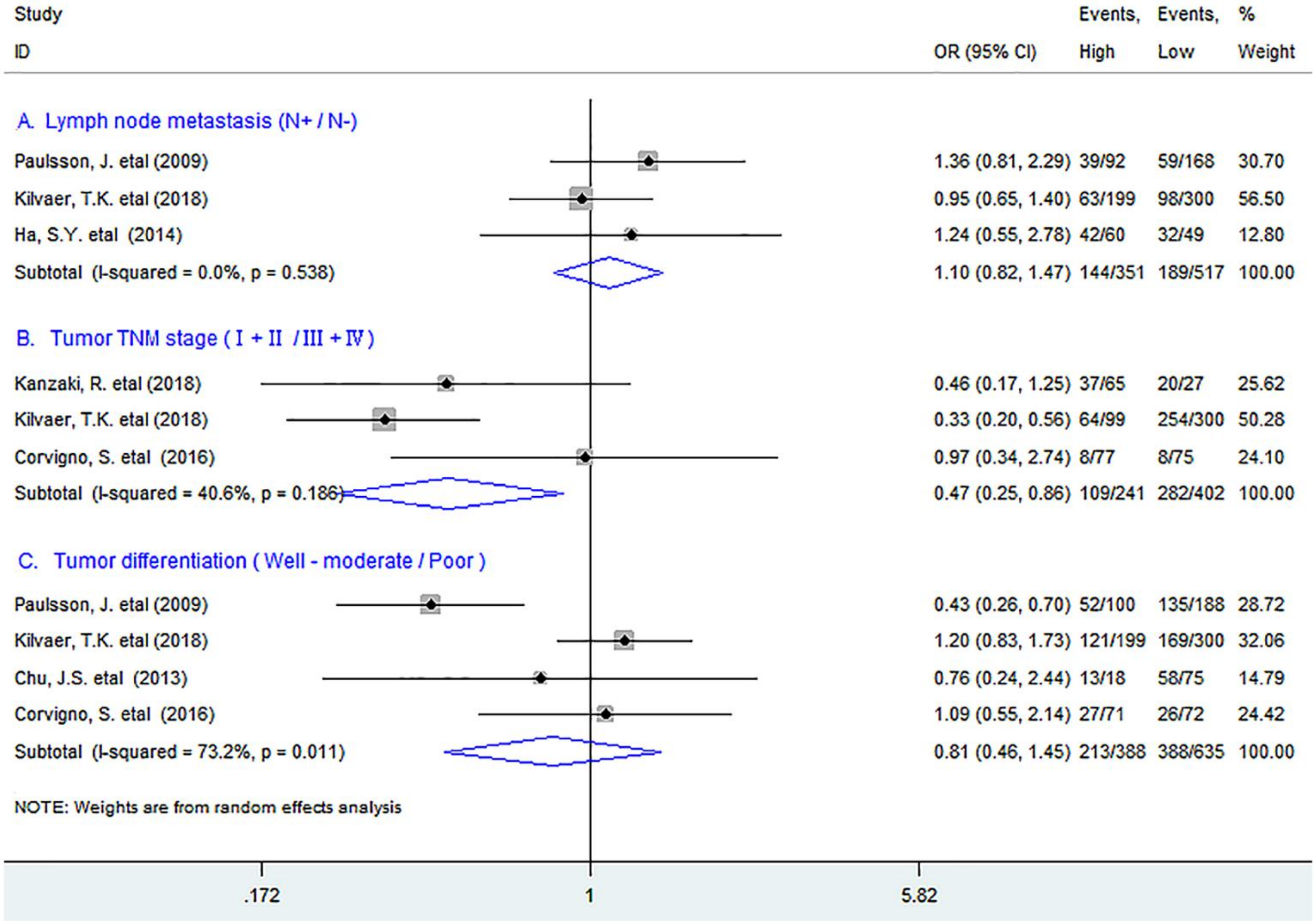

Supplementary Figure 2. Forest plots indicating ORs of the association between PDGFR-β+ fibroblast infiltration and clinicopathological features such as lymph node metastasis (**A**), tumor TNM stage (**B**), and tumor differentiation (**C**). ORs: odds ratios.

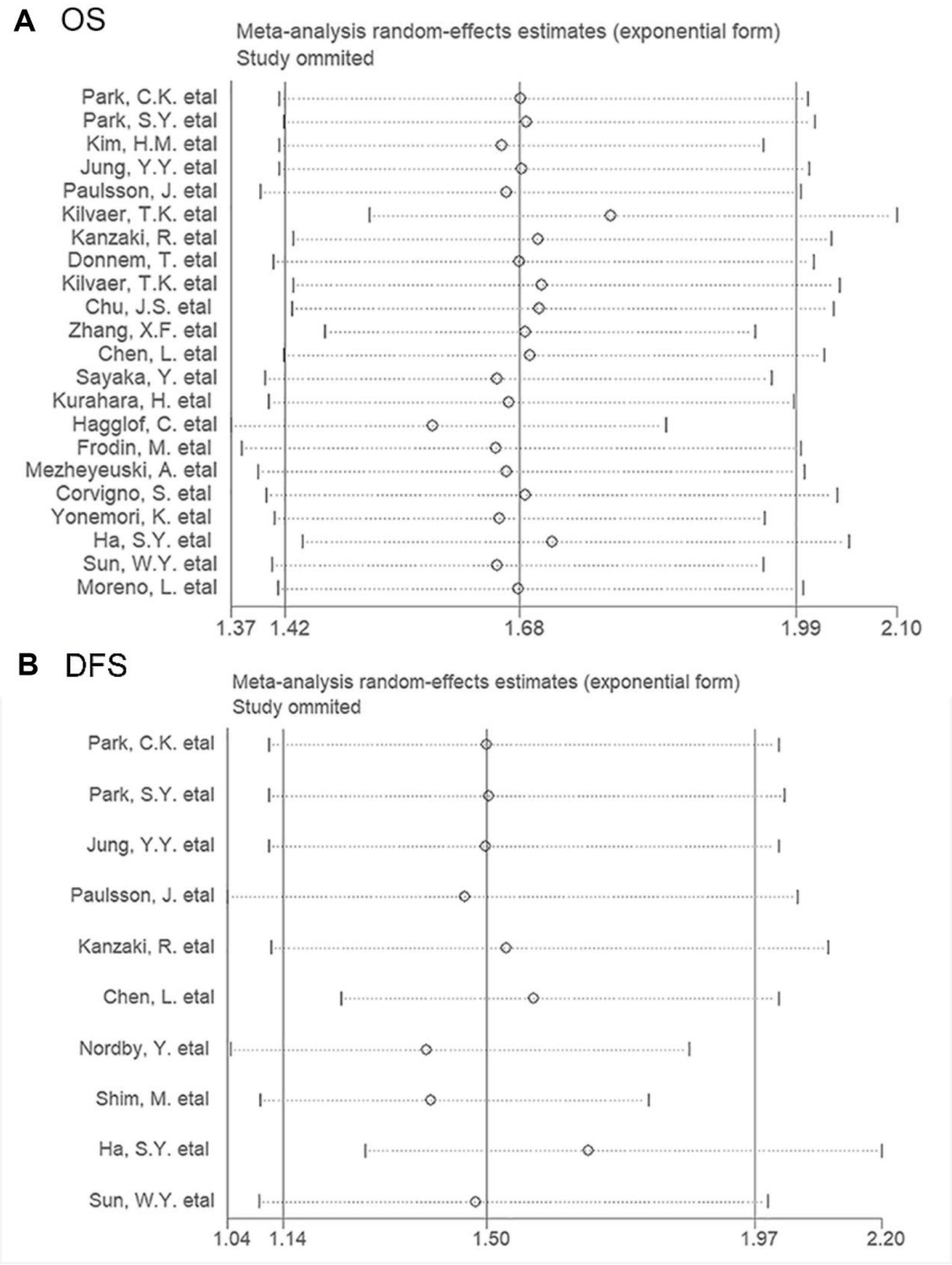

Supplementary Figure 3. Plots describing the influence of individual studies on the overall HRs for OS (**A**) and DFS (**B**) in solid tumors. HRs: hazard ratios; OS: overall survival; DFS: disease-free survival.

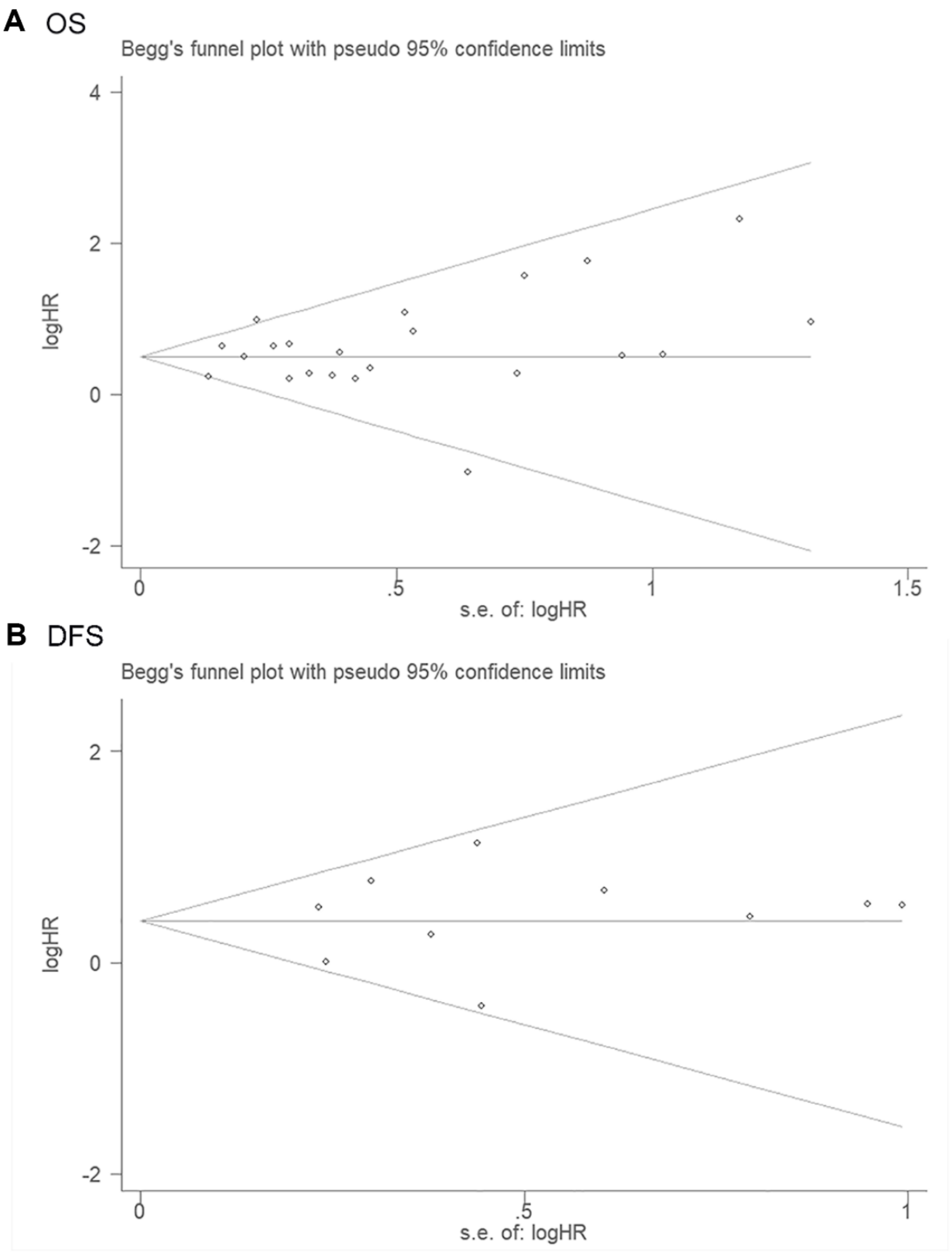

Supplementary Figure 4. Funnel plots for publication bias between PDGFR-β$^+$ fibroblasts and OS (**A**) and DFS (**B**) in solid tumors. OS: overall survival; DFS: disease-free survival.
