# Supplementary material for: PDGFR-β+ fibroblasts deteriorate survival in human solid tumors: a meta-analysis
Source: Aging (Albany NY). 2021 May 3;13(10):13693–707. doi: 10.18632/aging.202952 (PMC8202854; doi:10.18632/aging.202952)
Supplement: Supplementary Table 1 [file aging-13-202952-s002.pdf]

SUPPLEMENTARY TABLE

Supplementary Table 1. Characteristics of the included researches for OR analysis of clinicopathological features.

Research	Year	Tumor type	No. of patients	PDGFR-β⁺ fibroblasts: high/low	Lymph node metastasis (Yes / No)	Tumor stage (TNM)	I+II/III+IV	Tumor differentiation (well-moderate/poor)
Paulsson, J. etal [Paulsson et al., 2009]	2009	Breast cancer	289	100/189	H:(39/53); L:(59/109)	I - III	NR	H:(52/48); L:(135/53)
Kilvaer, T.K. etal [Kilvaer et al., 2018]	2018	NSCLC	499	199/300	H:(63/136); L:(98/202)	IA - IIIA	H:(64/35); L:(254/46)	H:(121/78); L:(169/131)
Ha, S.Y. etal [Ha et al., 2014]	2014	Esophageal squamous cell carcinoma	116	63/53	H:(42/18); L:(32/17)	I - IV	NR	NR
Kanzaki, R. etal [Kanzaki et al., 2018]	2018	NSCLC	92	65/27	NR	IA - IV	H:(37/28); L:(20/7)	NR
Corvigno, S. eta [Corvigno et al., 2016]	2016	Ovarian cancer	154	79/75	NR	I - IIIA	H:(8/69); L:(8/67)	H:(27/44); L:(26/46)
Chu, J.S. etal [Chu et al., 2013]	2013	Hepatic carcinoma	93	18/75	NR	III	NR	H:(13/5); L:(58/17)

N: lymph node; M: metastasis; H: high; L: low; NR: not reported.
